# A Retrospective Analysis of Incidental Gallbladder Cancer on Post-cholecystectomy Pathological Review

**DOI:** 10.7759/cureus.47249

**Published:** 2023-10-18

**Authors:** Murat Guner, Tayfun Kaya

**Affiliations:** 1 General Surgery, University of Health Sciences, Tepecik Training and Research Hospital, Izmir, TUR

**Keywords:** gallbladder malignancy, oncologic surgeries, incidental cancer, gallbladder cancer, incidental gallbladder cancer, cancer

## Abstract

Background

Gallbladder cancer is a rare cancer with a poor prognosis despite all the advances in treatment options and is mostly detected incidentally. In the current literature, re-excision is performed on patients with stage T1b and above, but high mortality rates are still observed. In this study, we aimed to investigate the reasons affecting the prognosis of incidental gallbladder cancer.

Methodology

Data from 33 patients were retrospectively analyzed. Patient age, sex, preoperative radiologic findings, surgical procedures, margin status, postoperative results with histologic diagnosis, T stage, complications, and mortality were evaluated.

Results

Of the 33 patients included in the study, 24 (72.7%) were female, nine (27.3%) were male, and the mean age was 66.4 ± 13.4 years. Seventeen (51.5%) patients in our study were aged over 65 years. Age over 65 years was found to have a significant effect on mortality (p = 0.018). In the preoperative ultrasound imaging, 27 (81.8%) patients had gallstones, two (6.1%) patients had gallbladder polyps, 31 (93.9%) had focal or diffuse thickness increases in the gallbladder wall, and nine (27.3%) patients had pericholecystic fluid. The presence of pericholecystic fluid (p = 0.039) and wall thickness (p = 0.006) were found to be associated with mortality. There was perineural invasion and lymphovascular in 17 patients each. Both perineural invasion (p = 0.016) and lymphovascular invasion (p = 0.007) were associated with mortality. Tumor grade was also associated with mortality (p = 0.001). When the prognosis of the patients was evaluated according to the T stage, the increase in the T stage negatively affected the prognosis (p < 0.001). Overall survival was a median of 17 months (95% confidence interval = 10.6-23.3).

Conclusions

Incidental gallbladder cancer is detected on routine histologic examination of gallbladder specimens after cholecystectomy. Most patients may require re-excision, but the prognosis is still poor in patients who have undergone re-excision. Age >65 years, pericholecystic fluid, T stage, grade, lymphovascular invasion, and perineural invasion had a significant effect on mortality, the presence of which should trigger the option of re-excision to be examined more carefully.

## Introduction

Gallbladder cancer (GBC), the fifth most common tumor of the gastrointestinal tract, is rare but has a high mortality rate [1]. Incidental GBC (IGBC) is detected on routine histologic examination of gallbladder specimens after cholecystectomy, and only a third of GBC patients have preoperative evidence of malignancy [2]. IGBC is usually asymptomatic and is difficult to diagnose in the preoperative period because it shows similar findings to other benign diseases of the gallbladder [3]. Most patients undergo preoperative ultrasound imaging, and increased wall thickness and irregularities of the wall are suspicious findings for malignancy, playing an important role in diagnosis [3]. Many causes are associated with the etiopathogenesis of the disease; gallstones, biliary polyps, obesity, age, and female sex are related to a higher incidence of GBC [4]. The only curative intent for GBC is surgery. Some patients can undergo surgery for emergency reasons in the advanced stage, and these patients are generally considered inoperable. Surgical procedures such as cholecystectomy and even duct resection can be performed palliatively in patients with unresectable disease [5]. One of the most important parameters that determine the extent of surgery is the T stage of the disease; therefore, in patients with IGBC, a second surgery may be required according to the T stage [6]. With appropriate surgical treatment, correct staging, and medical treatment, the five-year survival rate in stage pT1-2 disease can be up to 70%. On the other hand, in patients with lymphatic involvement or metastatic disease in advanced stages, the five-year survival rate ranges from 5% to 20% [1,7].

Surgeons should keep in mind that the disease may present with findings similar to benign gallbladder diseases in the early stages, and complicated wide surgical resections may be required in advanced disease. Although successful surgery is performed, advanced GBC shows high mortality rates [8]. In this study, we aimed to investigate whether IGBC had signs of malignancy in the preoperative radiologic imaging and the factors that affected survival.

## Materials and methods

Study design and ethical approval

Ethical approval for the current retrospective cohort study was obtained from the Ethical Committee of Non-invasive Clinical Research at the University of Health Sciences, Tepecik Training and Research Hospital on November 16, 2020 (approval number: 2020/13-35).

Variables

A total of 5,616 patients who underwent cholecystectomy between January 2008 and October 2020 were reviewed retrospectively. The data of 33 patients who were diagnosed with IGBC were analyzed. Patient age, gender, preoperative radiologic findings, surgical procedures, margin status, postoperative results with histological diagnosis, pathological T stage, complications, and mortality were evaluated retrospectively. Along with patients who underwent surgery with a provisional diagnosis of malignancy in our clinic, patients with missing data patients who were lost to follow-up were excluded from the study. Patients who underwent surgery for benign reasons were included in the study. Patients with a preoperative diagnosis of GBC or those with suspected GBC were excluded from the study. Finally, the data of 33 patients who were diagnosed with IGBC were included in the study.

Data were collected and recorded for patients’ demographic information, physical examination, radiologic imaging findings, the extent of surgical resection, histopathological findings, and survival.

Preoperative ultrasound was performed for all patients, and computed tomography (CT) and/or magnetic resonance imaging (MRI) were performed for patients with suspicious findings on ultrasound. Laparoscopic cholecystectomy was performed for all patients who underwent elective surgery. Open surgery was performed for patients who underwent emergency surgery. Five patients were Stage T1a and 1 patient was Stage T1b. The patient with Stage T1b disease on histopathologic examination refused reoperation; therefore, no patient with Stage T1 disease was reoperated. Therefore, re-excision was performed in patients with Stage T2 and T3 disease in whom the tumor was considered radiologically resectable. All patients were consulted by medical oncology, and adjuvant treatment was administered to patients with Stage T4 disease without re-excision. Abdominal CT or MRI was performed for all patients after the pathologic diagnosis of GBC was confirmed. All patients were consulted by a medical oncology specialist and called for regular outpatient follow-ups.

Statistical analysis

All statistical tests were performed using Microsoft Excel (Microsoft Corp., Redmond, WA, USA) and SPSS software version 22 for Windows (IBM Corp., Armonk, NY, USA). All continuous data were evaluated for normal distribution using the Shapiro-Wilk test, Q-Q plot, and histograms. Accordingly, when appropriate, continuous variables were presented as median (minimum-maximum) or mean ± standard deviation. The categorical variables were presented as frequency (percentage). Comparison of the continuous variables between the two groups was performed either through the Mann-Whitney U test or independent-sample t-test. The distribution of categorical variables between groups was compared using Pearson’s chi-square test or Fisher’s exact test. Multivariate analysis was performed via forward logistic regression analysis in which variables that revealed significant influence on mortality were added to the regression model. Survival analysis was performed using the Kaplan-Meier method, and the log-rank test was used to compare survival curves between the study groups. A p-value of less than or equal to 0.05 was considered statistically significant.

## Results

The mean age of the 33 patients in the study population was 66.4 ± 13.4 years. Seventeen (51.5%) patients in our study were aged over 65 years. Overall, 24 (72.7%) patients were female, and nine (27.3%) patients were male. As for the clinical data, while 16 (48.5%) patients had abdominal pain, four (12.1%) had jaundice, nine (27.3%) had acute abdomen, and four (12.1%) had abscess or fistula. Evaluation of the gallbladder revealed pericholecystic effusion/fluid in nine (27.3%), polyp in two (6.1%), and stone in 27 (81.8%) patients. While the mean wall thickness of the gallbladder and stone size were 9.4 ± 2.9 mm and 14.3 ± 6.8 mm, respectively, the median size of the polyp was 9 mm (8-10 mm). The median duration of follow-up was 17 (0-152) days. Overall, mortality was noted in nine (27.3%) patients.

Emergency surgery was performed on nine (27.3%) patients due to acute abdominal pain. Twenty-four (72.7%) patients underwent elective surgery. Re-excision was not performed on patients with Stage T4, Stage T1a, the Stage T1b patient who refused surgery. Re-excision was performed in patients with Stage T2 and Stage T3. Only cholecystectomy was performed in 14 (42.4%) patients, eight of whom were reoperated. Six (18.2%) patients with Stage T1 disease did not undergo a second surgery, and all of these patients were still alive at the time of evaluation. As the patient who had T1b disease refused second surgery, re-excision was performed in 22 patients with Stage T2 and Stage T3 disease. In advanced-stage disease, if the tumor was resectable, the surgery was planned to reach R0, and if it was unresectable, palliative surgery was performed in eligible patients. In the first surgery, cholecystectomy was performed in 14 (42.4%) patients, cholecystectomy + bed resection in 14 (42.4%) patients, and biopsy/partial cholecystectomy in five (15.2%) patients. Re-excision was performed in 22 patients whose histopathological examination revealed Stage T2 and Stage T3 disease. In the second surgery, hepatic segment 4b-5 resection + lymphadenectomy was performed in 10 (30.3%) patients, bile duct resection and choledochoenterostomy in seven (21.2%) patients, extended hepatectomy + lymphadenectomy in two (6.1%) patients, adjacent organ resection in two (6.1%) patients, and Whipple’s procedure in one (3.1%) patient. Lymphadenectomy was added to the surgery in all patients with resectable tumors. The surgical margin was positive in three patients, all of whom died. Among patients who needed a second surgery, Whipple’s procedure was performed in one patient, extrahepatic bile duct resection + choledochenterostomy was performed in one patient, and hepatic segment 4b-5 resection + lymphadenectomy was performed in one patient. The patient who underwent extrahepatic bile duct resection + lymphadenectomy and choledochenterostomy developed bile leakage on the fourth postoperative day. Drainage was performed but multiple organ dysfunction syndrome developed on the 30th postoperative day and the patient died. Intrahepatic metastases developed in the follow-up of the patient who underwent Whipple’s procedure, reoperation was not considered, and the patient died by the sixth-month follow-up. No postoperative complications were observed in the patient who underwent segment 4b-5 resection + lymphadenectomy, but local recurrence and intrahepatic metastasis developed in this patient and the patient died in the 18th month. According to the Clavien-Dindo classification, five (15.2%) patients developed grade 5 complications, five (15.2%) developed grade 3b complications, four (12.1%) developed grade 2 complications, and four (12.1%) developed grade 1 complications.

On univariate analysis (Table 1), the mean age of the patients with and without mortality did not reveal a significant difference (p = 0.190). While the rate of the patients, who were >65 years old, in the mortality group was higher than that of the patients without mortality, the difference was not significant (62.5% versus 22.2%, p = 0.057). The distribution of gender between the groups was not significant (p = 0.677). While the mean thickness of the gallbladder was significantly thicker in the mortality group (10.5 ± 2.4 mm versus 6.6 ± 2.1 mm, p < 0.001), the rate of pericholecystic fluid was higher in the mortality group (37.5 % versus 0%, p = 0.039).

**Table 1:** Demographic and clinical characteristics of the study population. ^α^: independent-sample t-test; ^β^: Fisher’s exact test; ^λ^: Mann-Whitney U test. * indicates mean ± standard deviation, and Φ indicates median (minimum-maximum) values.

			Mortality		
Variables		Overall (n = 33)	Yes (n = 24)	No (n = 9)	P-value
Age, years*		66.4 ± 13.4	68.3 ± 14.9	61.3 ± 6.1	0.190^α^
Age	>65 years	17 (51.5)	15 (62.5)	2 (22.2)	0.057^β^
	≤65 years	16 (48.5)	9 (37.5)	7 (77.8)	
Gender	Female	24 (72.7)	18 (75)	6 (66.7)	0.677^β^
	Male	9 (27.3)	6 (25)	3 (33.3)	
Abdominal pain		16 (48.5)	12 (50)	4 (44.4)	1.0^β^
Jaundice		4 (12.1)	4 (16.7)	0 (0)	0.555^β^
Acute abdomen		9 (27.3)	8 (33.3)	1 (11.1)	0.384^β^
Abscess/fistula		4 (12.1)	4 (16.7)	0 (0)	0.555^β^
Wall thickness, mm*		9.4 ± 2.9	10.5 ± 2.4	6.6 ± 2.1	<0.001^α^
Pericholecystic fluid		9 (27.3)	9 (37.5)	0 (0)	0.039^β^
Polyp		2 (6.1)	1 (4.2)	1 (11.1)	0.477^β^
Polyp size, mm^Φ^		9 (8–10)	8 (8–8)	10 (10–10)	0.317^λ^
Stone		27 (81.8)	20 (83.3)	7 (77.8)	1.0^β^
Stone size, mm*		14.3 ± 6.8	13.3 ± 5.9	17.4 ± 8.7	0.272^α^
Follow-up duration, days		17 (0–152)	13.5 (0–120)	86 (21–152)	<0.001^λ^

The characteristics of the malignancy are shown in Table 2. All patients had adenocarcinoma according to the histopathological examination. When the patients were examined according to the TNM stage, five (15.2% ) patients had T4 disease, 18 (54.5%) had T3 disease, two (6.1%) had T2b disease, two (6.1%) had T2a disease, one (1.3%) had T1b disease, and five (15.2%) patients had T1a disease. None of the patients in Stage T1 died during follow-up. The tumor was located at the corpus in 10 (30.3%), at the fundus in 19 (57.6%), and at both in four (12.1%) patients. While the mean size of the tumor was 26.2 ± 17.8 mm, it was higher in the mortality group; however, the difference was statistically not significant (30.5 ± 15.3 mm versus 14.7 ± 19.9 mm, p = 0.052). In the cohort, the rates of perineuronal and lymphovascular invasion were 51.5% and 51.5%, respectively. Additionally, the rates of perineuronal and lymphovascular invasion were significantly higher in the mortality group (66.7% versus 11.1%, p = 0.007 and 66.7% versus 11.1%, p = 0.007, respectively). While positive surgical margin did not have an influence on mortality (p = 0.545), the tumor grade (p < 0.001) and pathological T stage was considerably higher in the mortality group (p < 0.001).

**Table 2:** Univariate analysis of the characteristics of the malignancy and their effect on mortality. ^α^: independent-sample t-test; ^β^: Fisher’s exact test; ^λ^: Mann-Whitney U test. * indicates mean ± standard deviation, and Φ indicates median (minimum-maximum) values.

			Mortality		
Variables		Overall (n = 33)	Yes (n = 24)	No (n = 9)	P-value
Location	Corpus	10 (30.3)	8 (33.3)	2 (22.2)	0.792^β^
	Fundus	19 (57.6)	13 (54.2)	6 (66.7)	
	Both	4 (12.1)	3 (12.5)	1 (11.1)	
Tumor size, mm*		26.2 ± 17.8	30.5 ± 15.3	14.7 ± 19.9	0.052^α^
Perineuronal invasion		17 (51.5)	16 (66.7)	1 (11.1)	0.007^β^
Lymphovascular invasion		17 (51.5)	16 (66.7)	1 (11.1)	0.007^β^
Positive surgical border		3 (9.1)	3 (12.5)	0 (0)	0.545^β^
Tumor grade^Φ^		2 (1–3)	2 (1–3)	1 (1–2)	<0.001^λ^
Pathological T stage	1	6 (18.2)	0 (0)	6 (66.7)	<0.001^β^
	2	4 (12.1)	3 (12.5)	1 (11.1)	
	3	18 (54.5)	16 (66.7)	2 (22.2)	
	4	5 (15.2)	5 (20.8)	0 (0)	

On multivariate analysis, age >65 years (Exp(B) = 22.7, p = 0.018), lymphovascular invasion (Exp(B) = 52.9, p = 0.007), perineuronal invasion (Exp(B) = 16, p = 0.016), wall thickness (Exp(B) = 0.43, p = 0.006), and pathological T stage (Exp(B) = 24.5, p = 0.002) were found to have a significant effect on mortality in the model (Table 3).

**Table 3:** Multivariate analysis model of the variables on mortality.

Variables	B	Exp (B)	95% CI	P-value
Age >65 years	3.12	22.7	1.7–299	0.018
Lymphovascular invasion	3.97	52.9	2.95–948	0.007
Perineural invasion	2.77	16	1.7–151	0.016
Wall thickness	0.834	0.43	0.24–0.79	0.006
Pathological T stage (1–2 vs. 3–4)	3.20	24.5	3.37–178	0.002

**Figure 1:**
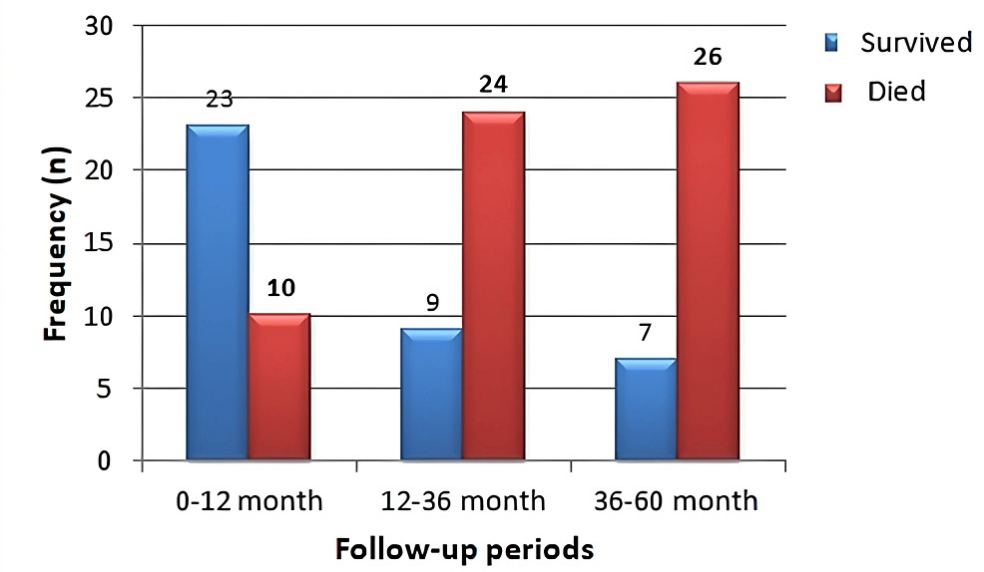
The distribution of survival and mortality within follow-up periods.

During the follow-up period, there were 10 (30.3%) deaths within the first 12 months, 24 (72.7%) within the 36 months, and 26 (78.8%) deaths at the end of 60 months. The distribution of survival and mortality is presented in Figure 1.

**Figure 2:**
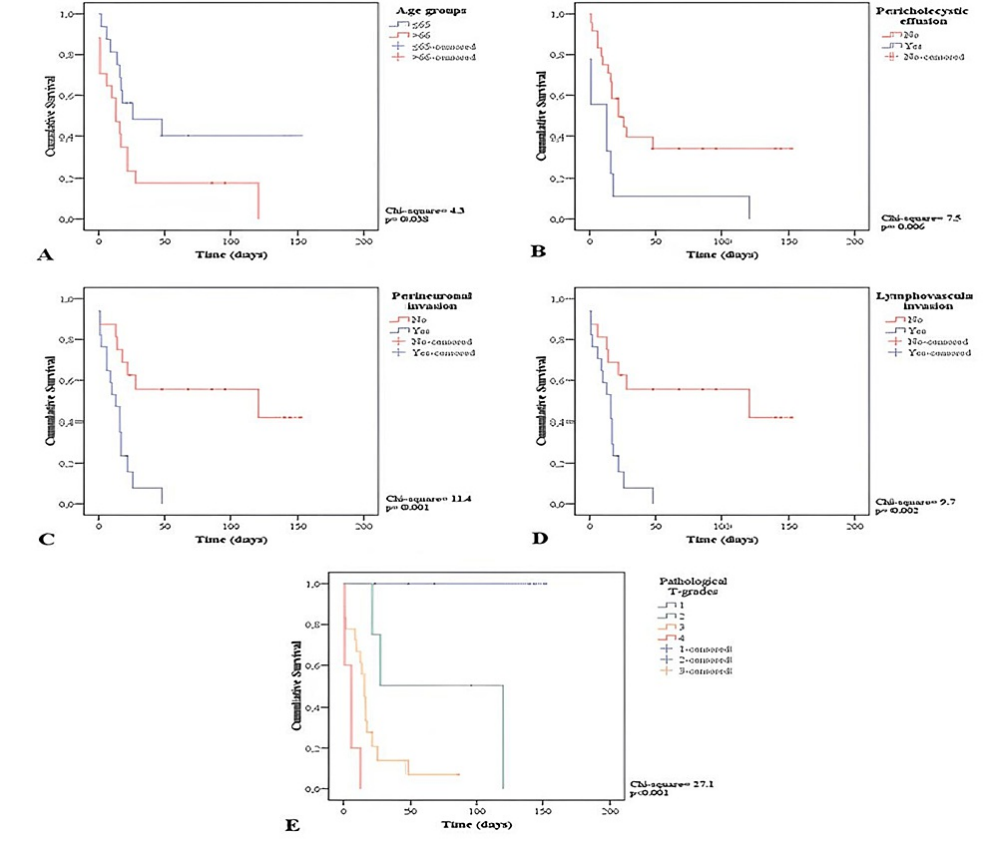
Kaplan-Meier survival curves showing the influencing variables on mortality. A: age groups. B: pericholecystic fluid/effusion. C: perineuronal invasion. D: lymphovascular invasion. E: pathological T stage.

On survival analysis (Figure 2), age >65 years (p = 0.038), pericholecystic fluid (p = 0.006), perineuronal invasion (p = 0.001), lymphovascular invasion (p = 0.002), and pathological T stage (p < 0.001) had a significant effect on mortality.

## Discussion

More than 50% of patients with GBC are diagnosed on pathology specimen examinations for benign reasons [9]. The only possible cure for GBC is surgery. Re-excision may be required in patients with incidentally detected GBC. In advanced disease, radical surgical resection may be required, and even if patients undergo surgery, the prognosis is not satisfactory.

Women and older patients are demographic risk factors for IGBC. In a study, the female-to-male ratio was 8:1. In our study, it was found to be 2.6:1. The mean age was 66.4 years, similar to the literature [10]. Age >65 years was found to have a significant effect on mortality.

A suspicious appearance may be detected on ultrasound or CT performed for other reasons, but it is difficult to diagnose during the preoperative period because benign gallbladder diseases can also show similar findings. Gallbladder stones are a risk factor for the disease and make it difficult to distinguish benign from malignant gallbladder disease at an early stage radiologically [11]. In our study, in the preoperative ultrasound imaging, 27 (81.8%) patients had gallstones, two had gallbladder polyps, 31 (93.9%) had focal or diffuse thickness increases in the gallbladder wall, and nine patients had pericholecystic fluid. Pericholecystic fluid may be due to gallbladder disease, as well as other organ pathologies and malignant diseases. Although the presence of pericholecystic fluid was found to be significant in this study, it should be investigated in more detail because it can also be seen in benign diseases of the gallbladder or of the peritoneum. Similarly, increased gallbladder wall thickness can also be seen due to benign causes; however, in our study, the presence of increased gallbladder wall thickness was found to have a significant effect on mortality. In the presence of pericholecystic fluid and increased gallbladder wall thickness on preoperative imaging, patients should be examined in more detail to differentiate malignancy. Tumor location may not be important in early-stage disease but may be important in advanced disease because of its potential to invade adjacent organs. In our study, the tumor location was the fundus in 19 (57.6%) patients, the corpus in 10 (30.3%) patients, and in the corpus and neck in four patients, but no significant difference was found.

Chen et al. stated that tumor differentiation affected prognosis in their study [12]. In our study, it was observed that grade affected mortality. The relationship between tumor grade and the prognosis appears to be a very important factor in the prognosis of tumor histopathology, as in other cancers.

In patients with GBC who undergo radical cholecystectomy with extrahepatic bile duct resection surgery, although direct invasion is not detected in the pathology specimen, GBC continues to spread by perineural and lymphatic routes. Perineural invasion has been reported to be a prognostic factor in GBC [13,14]. The presence of lymphovascular invasion is a poor prognostic factor in a wide range of tumor types [15]. In our study, the presence of lymphovascular invasion was also associated with poor prognosis.

The surgical margin was positive in three patients, all of whom died. All these patients needed a second surgery. Whipple’s procedure was performed in one patient, bile duct resection + choledochenterostomy was performed in one patient, and hepatic segment 4b-5 resection + lymphadenectomy was performed in one patient. Although all surgeons performing cancer surgery state that the positivity of surgical margins is a poor prognostic factor in patients with cancer, the degree of radicality required to achieve negative margins for early-stage tumors is not clear [16]. Although all three patients with positive margins died in our study, no significant correlation was found between surgical margin positivity and mortality. More studies with more patients are needed to determine the effect of surgical margin positivity on prognosis.

TNM is a frequently used staging system for patients with cancer, but the T stage is more commonly used to choose treatment for patients with GBC [17,18]. In this study, the increase in the T stage negatively affected the prognosis. Some surgeons have reported that margin-clearing surgery and lymphadenectomy might improve survival for patients with T1b disease [19,20]. However, in our study, re-excision was not performed on patients with T1a and the patient who refused re-excision with T1b disease. Surgery was performed by targeting the R0 surgical resection margin in patients with a disease stage higher than T1.

There are some limitations to this study. First, this is a retrospective study, and although the number of patients is sufficient for a rare tumor, more patients are needed for more detailed studies.

## Conclusions

GBCs are mostly discovered incidentally after routine cholecystectomy. Although many factors play a role in the choice of treatment, the option of re-excision remains controversial. In this study, we aimed to investigate the reasons that affected the prognosis of the patients. Age >65 years, presence of pericholecystic fluid, wall thickness, tumor grade, presence of lymphovascular invasion and perineural invasion, and T stage were seen as poor prognostic factors. In the presence of these factors, the option of re-excision should be examined more carefully.
